# Recapitulation of Human Embryonic Heartbeat to Promote Differentiation of Hepatic Endoderm to Hepatoblasts

**DOI:** 10.3389/fbioe.2020.568092

**Published:** 2020-09-08

**Authors:** Koki Yoshimoto, Nicolas Minier, Jiandong Yang, Satoshi Imamura, Kaylene Stocking, Janmesh Patel, Shiho Terada, Yoshikazu Hirai, Ken-ichiro Kamei

**Affiliations:** ^1^Institute for Integrated Cell-Material Sciences, Kyoto University, Kyoto, Japan; ^2^Department of Biosystems Science, Institute for Frontier Life and Medical Sciences, Kyoto University, Kyoto, Japan; ^3^Laboratory of Cellular and Molecular Biomechanics, Graduate School of Biostudies, Kyoto University, Kyoto, Japan; ^4^Department of Micro Engineering, Kyoto University, Kyoto, Japan; ^5^Department of Bioengineering, University of Pittsburgh, Pittsburgh, PA, United States; ^6^Department of Biomedical Engineering, University of Wisconsin–Madison, Madison, WI, United States; ^7^Wuya College of Innovation, Shenyang Pharmaceutical University, Shenyang, China; ^8^Department of Pharmacy, Shenyang Pharmaceutical University, Shenyang, China

**Keywords:** microfluidic device, human embryonic stem cells (hESC), heart beating, hepatic endoderm, hepatoblast, mechanical stimulation

## Abstract

Hepatic development requires multiple sequential physicochemical environmental changes in an embryo, and human pluripotent stem cells (hPSCs) allow for the elucidation of this embryonic developmental process. However, the current *in vitro* methods for hPSC-hepatic differentiation, which employ various biochemical substances, produce hPSC-derived hepatocytes with less functionality than primary hepatocytes, due to a lack of physical stimuli, such as heart beating. Here, we developed a microfluidic platform that recapitulates the beating of a human embryonic heart to improve the functionality of hepatoblasts derived from hepatic endoderm (HE) *in vitro*. This microfluidic platform facilitates the application of multiple mechanical stretching forces, to mimic heart beating, to cultured hepatic endoderm cells to identify the optimal stimuli. Results show that stimulated HE-derived hepatoblasts increased cytochrome P450 3A (CYP3A) metabolic activity, as well as the expression of hepatoblast functional markers (albumin, cytokeratin 19 and CYP3A7), compared to unstimulated hepatoblasts. This approach of hepatic differentiation from hPSCs with the application of mechanical stimuli will facilitate improved methods for studying human embryonic liver development, as well as accurate pharmacological testing with functional liver cells.

## Introduction

Hepatocytes are major components of the liver and have essential physiological roles, including protein and glucose synthesis and storage, detoxification, and excretion of exogenous molecules. Meanwhile, disruption of hepatic function can causes fibrosis, cirrhosis and even fatal liver cancers (Marengo et al., 2016; Altamirano-Barrera et al., 2017). Liver transplantation is currently the only effective curative method for these patients. Thus, new effective drug therapies are needed; however, drug discovery requires the use of primary hepatocytes to evaluate the toxicity of drug candidates prior to clinical trials (Bale et al., 2014). Meanwhile, identification of suitable healthy donors for liver transplantation, or hepatocytes for drug testing, remains challenging, warranting the development of alternative strategies for drug design and development.

One such strategy includes the use of human pluripotent stem cells (hPSCs), such as human embryonic and induced pluripotent stem cells [hESCs (Thomson et al., 1998) and hiPSCs (Takahashi et al., 2007; Yu et al., 2007), respectively], which show high potential due to their capacity for unlimited self-renewal and differentiation to almost any tissue cell type. Although many studies have been performed to obtain hPSC-derived hepatocytes, the resulting cells remain as immature hepatocytes with fewer functional properties. The cause of this limited development stems from the use of various biochemical factors, such as fibroblast growth factor (FGF), and bone morphogenesis protein (BMP) in the current differentiation protocols. These biochemical factors have been intensively investigated, however, the effects of biomechanical forces on the hepatic developmental process remain unknown. As mechanical forces regulate a variety of biological factors, including molecules, cells, tissues, and organs (Sakamoto et al., 2010; Ren et al., 2015), their effects must be considered to provide optimal hepatic differentiation methods from hPSCs.

Physiologically, hepatic endoderm (HE) is formed during the early developmental stages from definitive endoderm (DE), and HE-derived hepatoblasts give rise to hepatocytes or cholangiocytes (Gordillo et al., 2015). Notably, the HE is exposed to oscillating mechanical forces induced by the heart beating. We have hypothesized that such oscillating mechanical forces would influence the hepatic developmental process. However, although biochemical factors have been reported in static culture conditions (Si-Tayeb et al., 2010b; Camp et al., 2017), the effects of mechanical forces on the induction of HE differentiation into hepatoblasts have not yet been investigated due to limited access to human embryos, and the lack of proper *in vitro* models that recapitulate the physiological embryonic developmental process. Therefore, current protocols are incapable of evaluating the effects of mechanical forces induced by embryonic heart beating, and thus, result in immature differentiation of hepatocytes. Hence, more sophisticated platforms are required to address these shortcomings.

Microfluidic technology is a potential platform capable of applying mechanical forces to cells, as it allows for systematic manipulation of the cell-culture conditions (e.g., flow dynamics, cell-cell/matrix interactions, and mechanical stretching) in two- and three-dimensional models, which cannot be achieved using conventional cell-culture models. Recently, organs-on-a-chip platforms based on microfluidic technology have been reported to recapitulate physiological mechanical forces *in vitro* using natural tissues (Ho et al., 2006; Bhatia and Ingber, 2014; Kamei et al., 2017; Ronaldson-Bouchard and Vunjak-Novakovic, 2018). However, most organ-on-a-chip platforms can stimulate cells using only a single mechanical condition (Huh et al., 2010; Pavesi et al., 2015), and thus the optimal mechanical strength for obtaining targeted functional cells cannot be determined.

Here, we report a microfluidic platform for applying multiple mechanical forces to hPSC-derived HE cells to identify the optimal mechanical stress to facilitate differentiation of HE cells to functional hepatoblasts. The microfluidic device is composed of polydimethylsiloxane (PDMS) elastic material with a ballooned thin membrane as the cell-culture substrate, which can be actuated to mimic heart beating in an embryo. The balloons with cells inflate and deflate repeatedly via pressure regulation. We showed that hPSC-derived hepatoblasts differentiated under the optimal stretching condition and expressed enzymes of drug metabolism and proteins specific to hepatoblasts. These findings demonstrate that dynamic mechanical forces are critical for differentiation of HE to hepatoblasts and must, therefore be incorporated into differentiation models for hPSCs.

## Materials and Methods

### Chip Design

The formula for pressure drop, and length in microfluidic channel reported by Shimizu et al. (2011) was adapted. The molds for the pressure chambers and culture chambers were designed with a specific channel height (200 μm), and width (100 μm), and specific branch channel length (1 mm) by computer-aided design. The culture chambers were replaced considering the pressure drops.

### Computational Fluid Dynamics (CFD) Simulations

The air flow across the micro channel was simulated by COMSOL Multiphysics (Version 5.5, COMSOL Inc., Burlington, MA, United States) to calculate the pressure drop along the channel and chambers. The simulation was based on the coupling of the Navier-Stokes equation and the continuity equation in the stationary condition (Turgay and Yazıcıoğlu, 2018; Chen et al., 2019). The incompressible laminar flow was used to obtain the solutions. Due to the symmetrical structure, the geometric model of the right-half-side microfluidic channel and chambers were used to conduct the study. A physics-controlled extra fine mesh was used for dividing the domain grids. The flow media was set as the air material from the COMSOL material library. The boundary condition of the wall was set to no slip. The inlet pressure was set as the input pressure (i.e., 18, 32, and 45 kPa) with fully developed flow. The outlet port was assumed to be directly connected to the atmosphere condition and was set as the 0 pressure.

### Microfluidic Device Fabrication

A microfluidic device was fabricated using stereolithography 3D-printing techniques and solution cast-molding processes (Kamei et al., 2015). The molds for the top and bottom layer were produced using a 3D printer (Keyence Corporation, Osaka, Japan). After fabrication, the molds were washed with 99.9% EtOH for 12 h. The molds were dried at 80°C for 30 min. Sylgard 184 PDMS two-part elastomer (10:1 ratio of pre-polymer to curing agent; Dow Corning Corporation, Midland, MI, United States) was mixed, poured into a 3D-printed mold to produce a 5-mm-thick PDMS top layer and a 2-mm-thick PDMS bottom layer, and de-gassed using a vacuum desiccator for 30 min. The PDMS material was then cured in an oven at 80°C for 16 h. After curing, the PDMS form was removed from the mold, trimmed, and cleaned. Sylgard 184 PDMS two-part elastomer (10:1 ratio of pre-polymer to curing agent) was poured onto the silicon wafer, and spin-coated at 500 rpm for 30 s. After baking at 80°C for 10 min, the pressure chamber layer and the PDMS thin membrane on silicon wafer were treated with corona plasma (Kasuga Denki, Inc., Kawasaki, Japan), and subsequently placed at 80°C for 1.5 h for bonding. The bonded PDMS structure was peeled off the silicon wafer. The top layer-thin membrane forms, bottom layer, and glass were corona-plasma-treated and bonded together by placing in an oven at 80°C for 18 h. The devices were used within one day of completion.

### Device Control

The PDMS membranes was actuated by air flow from a compressed air resource (regulated at 0–200 kPa), operated with LabVIEW (Version 11.0, National Instrument, Austin, TX, United States) software via solenoid valves (Microfluidic System Works Inc., and THE LEE Company) using a controller board (VC3 8 controller [ALA Scientific Instruments] and NI USB-6501 [National Instruments]).

### Measurement of Pressure to Inlet and PDMS Displacement

The pressure was measured close to the inlet with a pressure sensor (ZS-46-5F; SMC, Tokyo, Japan). Vertical displacement of the PDMS thin membrane was measured with a CCD laser displacement sensor (LK-G5000; Keyence).

### hPSC Culture

hESCs were used according to the guidelines of the ethics committee of Kyoto University. H9 hESCs (WA09; **RRID:** CVCL_9773) were purchased from WiCell Research Institute (Madison, WI, United States). Prior to culture, hESC-certified Matrigel (Corning, Inc., Corning, NY, United States) was diluted with Dulbecco’s modified Eagle medium (DMEM)/F12 (Merck KGaA, Darmstadt, Germany) at a 1:75 (v/v) ratio and coated onto a culture dish. Matrigel was incubated in a dish for 24 h at 4°C. Excess Matrigel was removed, and the coated dish was washed with fresh DMEM/F12.

mTeSR-1-defined medium (Stem Cell Technologies, Vancouver, Canada) supplemented with 1% (v/v) penicillin/streptomycin (Fujifilm Wako, Osaka, Japan) was used for daily culturing of hPSCs. For passaging, the cells were dissociated with TrypLE Express (Thermo Fisher Scientific, Waltham, MA, United States) for 3 min at 37°C and harvested. The cells were centrifuged at 200 × g for 3 min, resuspended in mTeSR-1 medium and counted using Via 1-Cassete^TM^ (ChemoMetec A/S, Gydevang, 43, Denmark) of a NucleoCounter NC-200 (Chemetec, Baton Rouge, LA, United States). mTeSR-1 medium containing 10 μM of the ROCK inhibitor Y-27632 (Fujifilm Wako) was used to prevent apoptosis of dissociated hPSCs on day 1. mTeSR-1 medium without ROCK inhibitor was used on subsequent days, with daily medium changes.

### Hepatic Differentiation From hPSCs on Device

Prior to inducing differentiation, the culture chambers of the device were coated with Matrigel at 35°C for 60 min. Matrigel was removed with an aspirator.

To induce endoderm differentiation, cultured hPSCs were washed with D-phosphate-buffered saline (PBS) (no calcium, no magnesium) (Thermo Fisher Scientific) and treated with TrypLE Express at 37°C for 3 min, followed by addition of basal medium, and transfer of the cell suspension into a 15-mL tube. Cells were centrifuged at 200 × g for 3 min, after which the supernatant was removed. The cells were resuspended to 7.00 × 10^5^ cells mL^–1^ in mTeSR-1 medium supplemented with 10 μM Y27632, 100 ng mL^–1^ activin A (human recombinant) (Fujifilm Wako), and 1% (v/v) penicillin/streptomycin. They were then applied in 30 μL chambers^–1^ resuspended solution to a Matrigel-coated culture chambers and cultured in a humidified incubator at 37°C with 5% CO_2_ for 24 h. At the end of day 1, the medium was replaced with fresh mTeSR-1 medium supplemented with 10 μM Y27632, 100 ng mL^–1^ activin A and 1% (v/v) penicillin/streptomycin and cultured for an additional 24 h. On day 2, the medium was replaced with mTeSR-1 medium supplemented with 10 μM Y27632, 100 ng mL^–1^ activin A, 10 ng mL^–1^ BMP-4 (human recombinant) (R&D Systems, Minneapolis, MN, United States), 10 μM LY294002 (Cayman Chemical, Arbor, MI, United States), 3 μM CHIR99021(ReproCELL, Kanagawa, Japan), and 1% (v/v) penicillin/streptomycin. The cells were then incubated for 24 h. On day 3, the medium was replaced with mTeSR-1 medium supplemented with 10 μM Y27632, 100 ng mL^–1^ activin A, 10 ng mL^–1^ BMP-4, 10 μM LY294002, and 1% (v/v) penicillin/streptomycin, and the cells were incubated for 24 h. On day 4, the medium was replaced with Roswell Park Memorial Institute 1640 (RPMI) medium, GlutaMax Supplement (Thermo Fisher Scientific), supplemented with 2% (v/v) B-27 supplement (Thermo Fisher Scientific), 1% (v/v) MEM Non-essential Amino Acid Solution without L-glutamine, liquid, sterile-filtered Bioreagent suitable for cell-culture (NEAA) (Merck KGaA), 1% (v/v) penicillin/streptomycin, 10 μM Y27632, 100 ng mL^–1^ activin A, and 100 ng mL^–1^ bFGF (human recombinant) (Fujifilm Wako), and the cells were incubated for 24 h.

To induce HE specification, the cells were treated with RPMI medium GlutaMax Supplement, containing 2% (v/v) B-27 supplement, 1% (v/v) NEAA, 1% (v/v) penicillin/streptomycin, 10 μM Y27632 and 50 ng mL^–1^ activin A, with daily media changes for three days. On day 8, to induce hepatoblast specification, the cells were treated with RPMI medium GlutaMax^TM^ Supplement, containing 2% (v/v) B-27^TM^ supplement, 1% (v/v) NEAA, 1% (v/v) penicillin/streptomycin, 25 mM HEPS (Fujifilm Wako), 10 μM Y27632, 20 ng mL^–1^ BMP-4, and 10 ng mL^–1^ FGF-10 (human recombinant) (R&D Systems). The cells were then treated with RPMI medium GlutaMax^TM^ Supplement, containing 2% (v/v) B-27^TM^ supplement, 1% (v/v) NEAA, 1% (v/v) penicillin/streptomycin, 25 mM HEPES, 20 ng mL^–1^ BMP-4 and 10 ng mL^–1^ FGF-10, with daily media changes and 0.2 Hz mechanical stimulation for four days.

### Definitive Endoderm Differentiation on Dish

Prior to inducing differentiation, a cell-culture dish was coated with 0.1% gelatin from porcine skin, type A (Merck KGaA) in PBS at 25°C room temperature for 30 min. The gelatin solution was then aspirated and DMEM/F12 medium supplemented with 10% (v/v) fetal bovine serum (JRH Biosciences, St, Lenexa, KS, United States), 1% (v/v) L-glutamine, 1% (v/v) penicillin/streptomycin, and 100 μM b-mercaptoethanol (Fujifilm Wako) was introduced onto the culture dish for serum coating at 37°C for 24 h. The coated dish was then rinsed with fresh medium.

To induce endoderm differentiation, cultured hPSCs were washed with PBS and treated with TrypLE Express at 37°C for 3 min, followed by addition of basal medium and transfer of the cell suspension into a 15-mL tube. The cells were centrifuged at 200 × g for 3 min, after which the supernatant was removed. The cells were resuspended in mTeSR-1 medium supplemented with 1% (v/v) penicillin/streptomycin, 10 μM Y27632 and 100 ng mL^–1^ activin A, plated on a serum-coated culture dish, and cultured in a humidified incubator at 37°C with 5% CO_2_ for 24 h. After 24 h incubation on day 4, the cells were treated with RPMI medium, GlutaMax^TM^ Supplement, containing 2% (v/v) B-27^TM^ supplement, 1% (v/v) NEAA, 1% (v/v) penicillin/streptomycin, and 50 ng mL^–1^ activin A, with daily media changes for three days. The cells were then treated with RPMI medium, GlutaMax^TM^ Supplement, containing 2% (v/v) B-27^TM^ supplement, 1% (v/v) NEAA, 1% (v/v) penicillin/streptomycin, 20 ng mL^–1^ BMP-4 and 10 ng mL^–1^ FGF-10, with daily media changes for two days. On day 6, the cells were harvested for flow cytometry.

### Flow Cytometry

The cells were harvested with TrypLE Express and rinsed with PBS twice prior to cell counting. For antibody staining, the cells were diluted to a final concentration of 1 × 10^7^ cells mL^–1^ in staining buffer (fetal bovine serum) (BD PharMingen, Franklin Lakes, NJ, United States). 2 μL of fluorescence-labeled antibodies (APC mouse anti-human CD184 (CXCR4), clone 12G5; BD PharMingen) were added into 50 μL of cell suspension, and incubated at room temperature for 1 h. As a negative control, specific isotype controls (APC mouse IgG2a k isotype control, clone G155-178; BD PharMingen) were used with the same concentration of the primary antibody. After removing excess antibodies by centrifugation at 300 × g for 5 min, the cells were washed with staining buffer, and cell suspensions were applied to a FACS Canto II (BD Biosciences, Franklin Lakes, NJ, United States) for flow cytometric analysis. Data analysis was performed using FlowJo software (v9; FlowJo, LLC, Ashland, OR, United States).

### Cytochrome P450-GloTM Assay With Luciferin

To perform cytochrome P450-GloTM assays with luciferin (Promega, Madison, WI, United States), 20 μL medium was removed. Proluciferin IPA was diluted in RPMI medium, GlutaMax Supplement, containing 2% (v/v) B-27 supplement, 1% (v/v) NEAA, and 1% (v/v) penicillin/streptomycin. (1.5:1000). The air flow, mechanical stimulation was stopped, and 20 μL medium containing proluciferin IPA was applied to the culture chambers. The cells were incubated at 37°C with 5% CO_2_ for 1 h. Next, 25 μL × 2 medium containing luciferin IPA was collected from two culture chambers with the same mechanical stimulation and applied to the 96-well plates (White Microwell SI; Thermo Fisher Scientific); 50 μL P450-Glo Reagent was subsequently added, incubated at 28°C for 20 min, and relative light units were measured with a Synergy HTX Microplate Reader (Biotek, Winooski, VT, United States) at 28°C.

### Collection of all Proteins in Cells

The cells were rinsed with D-PBS (-) (Fujifilm Wako) three times, harvested with TrypLE Express, and collected into 1.5-mL tubes. Next, 0.5 mL RPMI medium, GlutaMax Supplement, containing 2% (v/v) B-27 supplement, 1% (v/v) NEAA, and 1% (v/v) penicillin/streptomycin was added. The tubes were centrifuged at 3000 × g for 3 min, and the supernatant was removed. The cells were rinsed with cold D-PBS, followed by addition of 100 μL cold 1 × RIPA buffer (Cell Signaling Technology, Danvers, MA, United States) in double distilled water was added. The tubes were then vortexed, incubated on ice for 30 min, sonicated at 4°C (US-1R cleaner; AS ONE, Osaka, Japan), and centrifuged at 4°C, 10,000 × g for 20 min. The supernatant was collected into new 1.5-mL tubes and stored at *−*20°C.

### Protein Quantification

Using a BCA protein kit (TaKaRa, Shiga, Japan), a working solution (BCA reagent A: B = 100:1) and 0.2 mg μL^−1^ BCA standard were mixed with the collected proteins in 100 μL 1 × RIPA buffer in a 96-well plate (Matrix Microplate w/lids 96-well blk/clr, flat bottom, tissue culture, PS; Thermo Fisher Scientific). Proteins were incubated at 37°C for 60 min and measured at a wavelength of 562 nm with a Synergy HTX Microplate Reader.

### Immunocytochemistry

The cells were fixed with 4% paraformaldehyde in D-PBS (-) (Fujifilm Wako) for 20 min at 25°C and then permeabilized with 0.1% (v/v) Triton X-100 in D-PBS for 5 min at 25°C. Subsequently, the cells were blocked in D-PBS (5% (v/v) normal goat serum blocking solution (Maravai Life Sciences, San Diego, CA, United States), 5% (v/v) normal donkey serum (Jackson ImmunoResearch, West Grove, PA, United States), 3% (v/v) albumin, essentially globulin-free (Merck KGaA), and 0.1% Tween-20 (Nacalai Tesque, Kyoto, Japan) at 4°C for 16 h and then incubated at 4°C for 16 h with primary antibodies [anti-human albumin mouse IgG, 1:500; R&D Systems: anti-human cytokeratin 19 (CK19) mouse IgG, 1:500; Thermo Fisher Scientific: anti-human cytochrome P450 3A7 (CYP3A7) rabbit IgG, 1:500; Proteintech, Chicago, IL, United States] in blocking buffer. The cells were then incubated at 37°C for 60 min with a secondary antibody (AlexaFluor 488 Donkey anti-mouse IgG (H + L), 1:1000; Jackson ImmunoResearch: AlexaFluor 594 Donkey anti-rabbit IgG (H + L), 1:1000) in 0.1% Tween-20 prior to a final incubation with 4’,6-diamidino-2-phenylindole (DAPI) (Fujifilm Wako) at 25°C for 30 min.

### Image Acquisition

The sample containing cells was placed on the stage of a Nikon ECLIPSE Ti inverted fluorescence microscope equipped with a CFI plan fluor 10 × /0.30 N.A. objective lens (Nikon, Tokyo, Japan), CCD camera (ORCA-R2; Hamamatsu Photonics, Hamamatsu City, Japan), mercury lamp (Intensilight; Nikon), XYZ automated stage (Ti-S-ER motorized stage with encoders; Nikon), and filter cubes for fluorescence channels (DAPI, GFP HYQ, TRITC; Nikon). For image acquisition, the exposure times were set to 200 ms for DAPI, 200 ms for GFP HYQ (for ALB), 50 ms for GFP HYQ (for CK19), and 200 ms for TRITC (for CYP3A7).

### Image Analysis

Image analysis of immunochemistry was performed with CellProfiler (ver. 3.1.9). The images of each stained protein and DAPI were inputted. In IdentifyPrimaryObejects, DAPI images were selected, and nuclei were identified. Typical diameter of objects, in pixel units was set (Min, Max) = (50, 150). Objects outside the diameter range and touching the border of image were discarded. Parameters were set as follows: Threshold smoothing scale, 6; Threshold correction factor, 0.95; Lower and upper bounds on threshold, 0.0 and 1,0; Size of adaptive window, 50; Method to distinguish clumped objects, shape; Method to draw dividing lines between clumped objects, shape. Size of smoothing filter for declumping, and minimum allowed distance between local maxima were automatically calculated. Handling was sped up by using lower-resolution images to identify local maxima. Holes in identified objects were filled only after declumping. Handling of objects was continued if an excessive number of objects was identified. In IdentifySecondaryObjects, stained protein images were selected, and nuclei, as the input objects, was selected. The parameters were set as follows: Method to identify the secondary objects, Propagation; Threshold strategy, Global; Thresholding method, Minimum cross entropy; Threshold smoothing scale, 0.0; Threshold correction factor, 1.0; Lower and upper bounds on threshold, 0.0 and 1.0; Regularization, 0.05. Holes were filled in identified objects. Objects touching the border of images were not discarded. In IdentifyTertiaryObjects, as the larger identified objects, secondary identified objects were selected, and as the smaller identified objects, primary identified objects were selected. Prior to subtraction, smaller objects were not shrunken. In MeasureObjectIntensity, the intensity of tertiary identified objects, cytoplasm, was measured.

### Statistical Analysis

Each sample was labeled alphabetically. The Tukey-Kramer test was carried out with *R* software (ver. 4.0.2).

## Results

### Fabrication of a Microfluidic Device Mimicking the Beating of an Embryonic Heart

**FIGURE 1:**
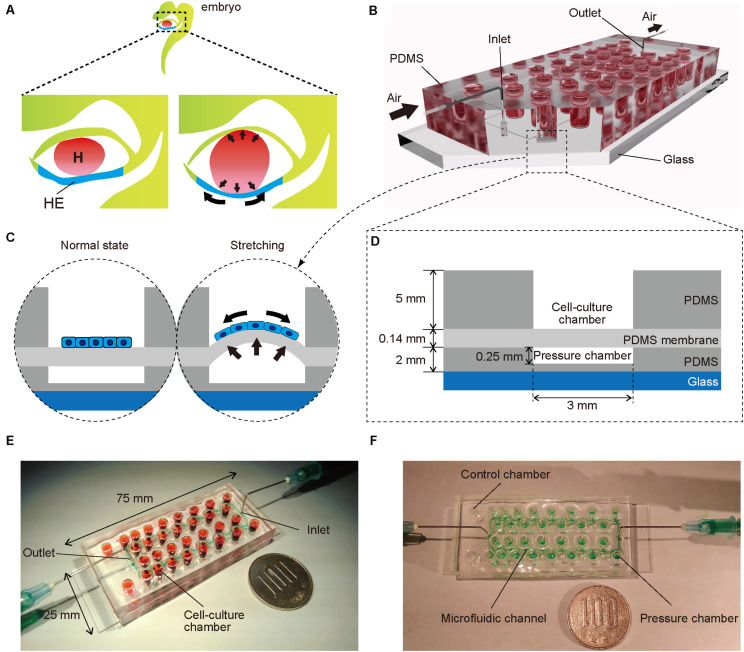
**(A)** Illustration of an early human embryo. Heart (H: red) beating confers mechanical stimulation to the surrounding cells, and hepatic endoderm (HE: blue), which differentiates to hepatoblasts, is exposed to mechanical forces. **(B,C)** Appearance **(B)** and cross-section **(C)** of a microfluidic device for applying a series of stretching stimulations to hepatic endoderm-like cells (HECs). **(D)** Cross-sectional view of the device, composed of polydimethylsiloxane (PDMS) and consists of a top layer with cell-culture chambers, middle membrane layer, and bottom layer with pressure chambers on a glass slide. **(E)** Photograph of our device fabricated on a glass slide (25 × 75 mm). Culture chambers are filled with red ink. This device has two sets of culture chambers in which cells are cultured under the same intensity of mechanical stimulation. **(F)** Photograph of a microfluidic device. Microfluidic channels and pressure chambers filled with green ink.

To mimic embryonic heart beating *in vitro* (Figure 1A), a microfluidic device with a series of stretchable balloon membranes was fabricated (Figures 1B–F and Supplementary Figure S1). This microfluidic device consisted of three layers: a top layer for cell-culture wells, middle layer of thin membrane as the stretchable cell-culture substrate, and bottom layer for forming pressure chambers. The top layer was 5 mm thick, and each well in the top layer was 3 mm in diameter. The middle PDMS membrane was 0.14 mm thick. The bottom layer was 2 mm thick with a 0.25-mm channel and chamber height, and 0.2-mm channel width (Supplementary Figure S2). The molds for the top and bottom layers were fabricated with a high-resolution 3D printer (Kamei et al., 2015).

**FIGURE 2:**
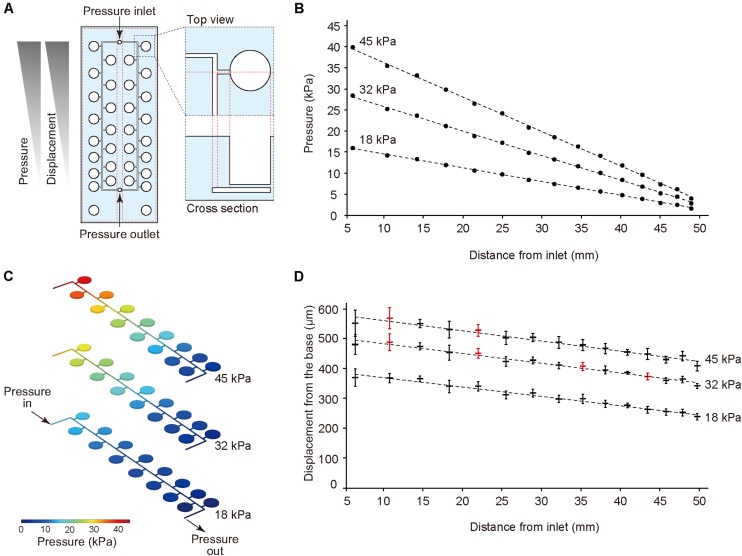
Pressure-drop method to generate a series of PDMS membrane displacements in a single device. **(A)** Membrane displacements are inversely proportional to the distance from the inlet due to a pressure drop along with a microfluidic channel. **(B,C)** The computer simulation for pressure drop was conducted using input pressure (18, 32, and 45 kPa) and output pressure (atmosphere). **(D)** Displacement measurement of the device with CCD laser displacement camera when 18, 32, and 45 kPa were applied at the inlet. The red plots indicated the conditions used for stretching stimulation. ANOVA with Tukey-Kramer test compared with all displacements of the pressure chambers at 18, 32, and 45 kPa. *p*-values were summarized in Supplementary Tables S1, S2. Each plot represents the mean ± standard deviation determined from four independent experiments measuring the two chambers in a single device.

The thin ballooned PDMS membrane was actuated with the regulator connected to an air compressor. To test a series of stretching forces within a single device, we used a pressure-drop method (Shimizu et al., 2011) in which air pressure was decreased in an inverse proportion to the length of fluidic flow (Figure 2A). Hence, the pressure drop (Δ*P*) for incompressible fluid flow was determined from the Fanning friction factor (f) using the Fanning formula:

(1) $$\Delta P=4f\left(\frac{L}{D}\right)\left(\frac{\rho v^{2}}{2}\right)$$

Where *L* is the channel length, ρ is the fluid density, and *v* is the average velocity in a channel. Hydraulic diameter, D (m) was calculated as:

(2) $$D=\frac{2wh}{\left(w+h\right)}$$

where *w* and *h* represent the channel width and height, respectively.

The amount of air pressure applied to each chamber decreased with increasing channel length. The device was designed to have two sets of 15 culture chambers along a micro channel and negative control chambers in a single device. For the CFD simulation of pressure drop in the device, incompressible flow and laminar flow in microscale channel was considered, and three input pressures (i.e., 18, 32, and 45 kPa), as well as the output pressure (atmosphere) were set. The analytical simulation results showed linear damping characteristic curves of pressures similar to theoretical equation (1) (Figures 2B,C). To demonstrate the pressure-drop method over a series of membrane stretching events with the actual device, we applied three input pressures (i.e., 18, 32, and 45 kPa) to the inlet and measured the vertical displacement from the base of the membrane. As expected, membrane displacement corresponded to pressure decreases for the tested input pressures. When more than 45 kPa pressure was applied to the inlet, the device was unstable due to air leakage. Based on these results, we selected seven significant different displacements and labeled them as the input pressure (kPa), and the distance from the inlet (mm), such as (45, 10.4) (Figure 2D, Supplementary Tables S1, S2).

### Differentiation of hPSCs to Hepatoblasts

**FIGURE 3:**
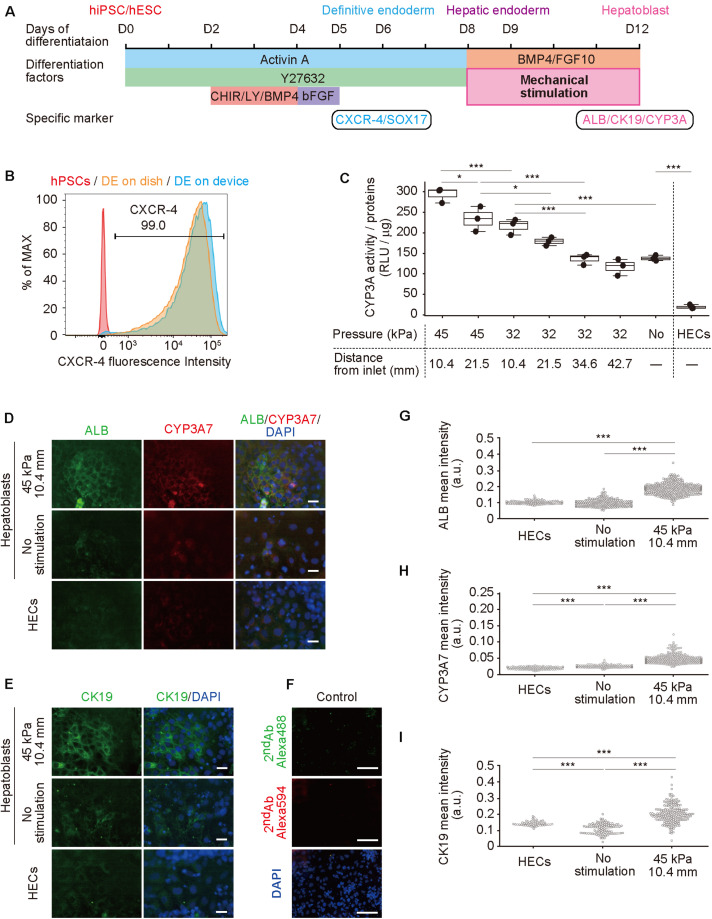
Hepatoblast differentiation from human pluripotent stem cells (hPSCs) promoted by mechanical forces on hepatic endoderm-like cells (HECs). **(A)** Schematic diagram showing hepatic differentiation from hPSCs. ROCK inhibitor (Y27632), WNT inhibitor (CHIR, CHIR99021), PI3K inhibitor (LY, LY294002), bFGF, BMP4, and FGF10 were used for corresponding differentiation stages. **(B)** Flow cytometric analyses showing the proportion of CXCR4 expression in hPSCs, HECs in a dish and HECs in a microfluidic device. **(C)** Bioluminescent CYP3A activity assay for HECs and hepatoblasts. ANOVA with Tukey-Kramer test compared relative light units (RLU) divided by amount of proteins in cell lysates of all samples (Supplementary Figure S6 and Supplementary Table S3). *P < 0.05, ***P < 0.001 (*n* = 3). Max, median, minimum of three independent experiments were shown. **(D,E)** Immunocytochemical analyses showing the expression of ALB, CYP3A7, **(D)** and CK19 **(E)** in HECs and hepatoblasts in the indicated conditions. Nuclei were stained with DAPI. Scale bars represent 50 μm. **(F)** The negative controls of immunochemistry stained with only 2nd antibody labeled with Alexa 488 and Alexa 594 fluorescent dyes shown in **(F)**. Scale bars represent 100 μm. **(G–I)** Quantitative single cell profiling of ALB **(G)**, CYP3A7 **(H)**, and CK19 **(I)** in HECs and hepatoblasts in the device. Fluorescence intensity was obtained from several images per each sample. ANOVA with Tukey-Kramer test compared with mean intensity. ***P < 0.001.

Next, hPSCs were differentiated to hepatoblasts in a device (Figure 3A; Hannan et al., 2013; Kamei et al., 2019). In our previously reported protocol using a dish, gelatin coating was sufficient for hPSC culture and differentiation, however, was insufficient for PDMS membrane, causing detachment of cells (Supplementary Figure S3). Therefore, prior to culturing in a device, the cell-culture chambers were coated with Matrigel, which improved hPSC adhesion and differentiation. The differentiation from hPSCs to hepatoblasts occurred via three stages. Briefly, in the first stage, hPSCs were directed into DE by treatment with 100 ng mL***^–^***^1^ activin A, 10 μM ROCK inhibitor, 3 μM CHIR99021, 10 μM LY294002, 10 ng mL***^–^***^1^ BMP4, and 100 ng mL***^–^***^1^ basic FGF (bFGF). To confirm DE differentiation, expression of C-X-C-motif chemokine receptor 4 (CXCR4), a DE cell-surface marker, as well as SRY-box 17 (SOX17), a DE transcription factor (D’Amour et al., 2005; Yasunaga et al., 2005), were evaluated by flow cytometry (Figure 3B) and immunocytochemistry (Supplementary Figure S4) at day 6. More than 99% of the cells were stained for CXCR4, and also showed expression of SOX17, indicating efficient DE differentiation from hPSCs. In the second stage, DE cells were treated with a lower concentration of activin A (50 ng μL***^–^***^1^) to obtain hepatic endoderm-like cells (HECs). In the third stage, HECs were differentiated to hepatoblasts by treatment with 20 ng mL***^–^***^1^ BMP4 and 10 ng mL***^–^***^1^ FGF10. Frequency of stretching stimulation, set at 0.2 Hz (Supplementary Video S1), was applied during the third stage for four days. Cells were observed on the PDMS thin membrane by day 12 (Supplementary Figure S5 and Supplementary Video S2).

### Mechanical Forces Enhance Differentiation of hPSCs to Functional Hepatoblasts

To investigate the effect of mechanical forces on the differentiation of HECs to hepatoblasts, the activity of cytochrome P450 3A (CYP3A), which is specifically expressed in hepatic cells (Si-Tayeb et al., 2010b; Danoy et al., 2020), was measured in a bioluminescent CYP3A activity assay at day 12 (Figure 3C, Supplementary Figure S6 and Supplementary Table S3). Compared with HECs induced on the device, unstimulated hepatoblasts showed significantly higher activity, as expected. When 32-kPa input pressure was applied to the larger displacements at (32, 21.5) and (32, 10.4), stimulated hepatoblasts showed higher CYP3A activity, whereas activity at (32, 42.7) and (32, 34.6) was not significantly different from that observed for unstimulated haptoblasts. Moreover, at a higher input pressure of 45 kPa, the larger displacements at (45, 21.5) and (45, 10.4) caused significantly higher CYP3A activity in hepatoblasts compared to unstimulated cells, and hepatoblasts at (45, 10.4), which had at least two-fold higher CYP3A activities compared to unstimulated cells. These results suggest that mechanical stimulation, induced by stretching cell-culture substrates, increased hepatoblastic metabolic activities.

To further investigate the effects of stretching stimulation on HEC-hepatoblast differentiation, the expression of albumin (ALB), CYP3A7, and cytokeratin 19 (CK19) proteins, which are specifically expressed in hepatoblasts (Schmelzer et al., 2006; Yang et al., 2017), were observed by immunocytochemistry (Figure 3D for ALB and CYP3A7, Figure 3E for CK19, Figure 3F for negative controls stained with only 2^nd^ antibodies and Supplementary Figure S7 for positive control with HepG2 cells). The fluorescence intensity of the single cell was calculated (Figure 3G for ALB, Figure 3H for CYP3A7, Figure 3I for CK19). Hepatoblasts at (45, 10.4) showed higher expression of ALB, CYP3A7, and CK19 proteins than unstimulated hepatoblasts, which agreed with the results observed for CYP3A activities. Notably, HECs did not express any of the examined proteins, and no secreted albumin was detected in the conditioned media (Supplementary Figure S8). Generally, these proteins have only been shown to be expressed by hepatoblasts *in vivo*, not *in vitro* (Si-Tayeb et al., 2010b; Danoy et al., 2020). Hence, these results suggest that application of stretching stimulation induces more functionally relevant hPSC-derived hepatoblasts compared to those generated via conventional cell-culture methods.

## Discussion

Herein, we recapitulated human embryonic development from hESC-derived HE to hepatoblasts with mechanical stimulation to improve functionality of hepatocytes. Until now, most hepatic differentiation methods for hPSCs are based on treatments with biochemical substances, such as growth factors, cytokines, supplemental chemicals, and extracellular matrices. Although these methods allow for the generation of hPSC-derived hepatocyte-like cells, they do not facilitate the development of fully functional hepatocytes. Hence, methodological developments require a new approach for the differentiation of hPSCs. To this end, we hypothesized that mechanical stimulation, such as the stretching force generated by a heart beating, would influence hepatoblast functionality during hepatic differentiation.

Results from our newly developed model showed that ALB expression of stimulated hepatoblasts at (45, 10.4) was higher than that of unstimulated hepatoblasts. Hence, GATA4, which is reportedly an enhancer of ALB, may be susceptible to mechanical stimulation thorough RhoA (Charron et al., 2001; Si-Tayeb et al., 2010a). Moreover, the expression of CYP3A7 and CK19 was also higher in hepatoblasts at (45, 10.4) compared to unstimulated hepatoblasts. According to Genecards (Zhang et al., 2018), which provides information on genes and their expression mechanisms, the enhancer of CYP3A7 and CK19 is peroxisome proliferator-activated receptor-γ (PPARg), which serves as a co-activator with the transcription factors yes-associated protein (YAP), and transcriptional co-activator with PDZ-binding motif (TAZ), in the Hippo pathway (Piccolo et al., 2014). Recently, it was reported that mechanical stimuli via cell-substrate interactions induces the translocation of YAP/TAZ, and alters the phenotype of epithelial (Acharya et al., 2018), endothelial (Sakamoto et al., 2010; Nakajima et al., 2017), and mesenchymal stem cells (Hong et al., 2005; Dupont et al., 2011; Guo et al., 2018). This process may, therefore, be involved in HEC-hepatoblast differentiation. However, the precise underlying mechanisms associated with this pathway require further investigation to provide specific improvements to the mechanical stimulation of HEC-hepatoblast differentiation in the future.

The use of hPSC-derived hepatocytes has been proposed as an alternative to human primary hepatocytes for drug screening (Bale et al., 2014; Zakikhan et al., 2017); however, no previous studies have described methodology capable of generating cells with the proper functionality, due to insufficient differentiation. Our methodology designed to obtain functional hepatoblasts will allow the generation of more functional hepatocytes that will be directly applicable to Liver-on-a-Chip technologies, for applications in drug screening and toxicological testing (Kamei et al., 2017; Ronaldson-Bouchard and Vunjak-Novakovic, 2018). Furthermore, functional hepatocytes can be used for various *in vitro* disease modeling, including infection (Nie et al., 2018) and non-alcoholic fatty liver disease (Nantasanti et al., 2015; Lauschke et al., 2019). In addition to the *in vitro* applications, since the hepatocytes generated from our device can be harvested, they may also prove applicable for cell transplantation and regenerative medicine (Prior et al., 2019). Therefore, our novel method for the generation of functional hepatoblasts from hPSCs may prove to be a novel platform for both *in vitro* and *in vivo* applications of hPSC-derived hepatocytes.

Certain limitations were noted in this study. For instance, the frequency of stretching stimulation applied in our system was 0.2 Hz, equivalent to 12 beats per minute, which is much slower than that of normal fetal heart rates (110 to 150 beats per minutes) (Pildner von Steinburg et al., 2013). This is largely due to the current experimental setting to actuate the PDMS membrane using the solenoid valve, which is not capable of achieving normal fetal heart rate frequencies. Hence, to truly mimic fetal liver developmental conditions, more rapid stretching stimulation of cells must be applied.

## Conclusion

In summary, we developed a microfluidic device by applying multiple stretching forces during HEC-hepatoblast differentiation from hPSCs. Using this device, we found that mechanical stimulation improved the functionalities of hepatoblasts. Our device and approach provide not only insights into the hepatic developmental process, but also tools for applications in both drug discovery and regenerative medicine.

## Data Availability Statement

The authors acknowledge that the data presented in this study must be deposited and made publicly available in an acceptable repository, prior to publication. Frontiers cannot accept a manuscript that does not adhere to our open data policies.

## Author Contributions

KY, NM, and KK performed conceptualization, investigation, and methodology. KY, NM, JY, SI, KS, JP, ST, and YH performed data curation. KY, NM, JY, and YH performed formal analysis. KK performed funding acquisition, project administration, resources, and supervision. YH and KK performed software. KY and KK performed validation and visualization and writing original draft. KY, NM, JY, KS, JP, YH, and KK wrote, reviewed and edited the manuscript. All authors contributed to the article and approved the submitted version.

## Conflict of Interest

The authors declare that the research was conducted in the absence of any commercial or financial relationships that could be construed as a potential conflict of interest.
