# Supplementary material for: Recapitulation of Human Embryonic Heartbeat to Promote Differentiation of Hepatic Endoderm to Hepatoblasts
Source: Front Bioeng Biotechnol. 2020 Sep 8;8:568092. doi: 10.3389/fbioe.2020.568092 (PMC7506096; doi:10.3389/fbioe.2020.568092)
Supplement: Supplementary file 1 [file Data_Sheet_1.docx]

Supplementary Material

Recapitulation of human embryonic heartbeat to promote differentiation of hepatic endoderm to hepatoblasts

Koki Yoshimoto^1,2,3^, Nicolas Minier^1^, Jiandong Yang,^4^ Satoshi Imamura^1^, Kaylene Stocking^1,5^, Janmesh Patel^1,6^, Shiho Terada^1^, Yoshikazu Hirai,^4^ Ken-ichiro Kamei^1,7,8^*

^1^Institute for Integrated Cell-Material Sciences, Kyoto University, Yoshida-Ushinomiya-cho, Sakyo-ku, Kyoto 606-8501, JAPAN.

^2^Department of Biosystems Science, Institute for Frontier Life and Medical Sciences, Kyoto University, Shogoin-Kawara-cho, Sakyo-ku, Kyoto 606-8397, JAPAN

^3^Laboratory of Cellular and Molecular Biomechanics, Graduate School of Biostudies, Kyoto University, Yoshida-Konoe-cho, Sakyo-ku, Kyoto 606-8397, JAPAN

^4^Department of Micro-Engineering, Kyoto University, Kyotodaigaku-Katsura, Nishikyo-ku, Kyoto 616-8540, JAPAN

^5^Department of Bioengineering, University of Pittsburgh, Pittsburgh, PA 15260, USA

^6^Department of Biomedical Engineering, University of Wisconsin Madison, Madison, WI 53706, USA

^7^Wuya College of Innovation, Shenyang Pharmaceutical University, Liaoning 110016, China

^8^Department of Pharmaceutics, Shenyang Pharmaceutical University, Liaoning 110016, China

*** Correspondence:**Ken-ichiro Kamei
kamei.kenichiro.7r@kyoto-u.ac.jp

# Supplementary Methods

## Scanning electron micrograph (SEM)

## A 5-nm-thick platinum film was deposited on the device sectioned with a knife by sputtering (MSP 30T; Shinku Device, Ibaraki, Japan). Images of the device were acquired with a scanning electron microscope at 10 kV (JCM-5000; JEOL Ltd., Tokyo, Japan).

## Immunocytochemistry of SOX17

The cells were fixed with 4% paraformaldehyde in D-PBS (-) (Fujifilm Wako) for 20 min at 25 °C and then permeabilized with 0.1% (v/v) Triton X-100 in D-PBS for 16 h at 25 °C. Subsequently, the cells were blocked in D-PBS (5% (v/v) normal goat serum blocking solution (Maravai Life Sciences, San Diego, CA, USA), 5% (v/v) normal donkey serum (Jackson ImmunoResearch, West Grove, PA, USA), 3% (v/v) albumin, essentially globulin-free (Merck KGaA), and 0.1% Tween-20 (Nacalai Tesque, Kyoto, Japan) at 4 °C for 16 h, and then incubated at 4 °C for 16 h with the primary antibody [anti-human SOX 17 mouse IgG, 1:100; R&D Systems] in blocking buffer. The cells were then incubated at 37 °C for 60 min with a secondary antibody (AlexaFluor 488 donkey anti-mouse IgG (H+L), 1:1000; Jackson ImmunoResearch) in 0.1% Tween-20 prior to a final incubation with 4′,6-diamidino-2-phenylindole (DAPI; Fujifilm Wako) at 25 °C for 30 min.

## HepG2 cell culture on a dish

HepG2 human hepatocellular carcinoma cell line was obtained from the American Type Culture Collection. Cells were maintained in Dulbecco’s modified Eagle medium (DMEM; Sigma-Aldrich) supplemented with 10% (v/v) fetal bovine serum (FBS; Cell Culture Bioscience), 1% (v/v) nonessential amino acids (Thermo Fisher Scientific), and 1% (v/v) penicillin/streptomycin (Thermo Fisher Scientific) in a humidified incubator at 37 °C with 5% (v/v) CO_2_. HepG2 cells were passaged with trypsin/EDTA (0.04%/0.03%[v/v]) solution every three days at ratios of 1/5.

## HepG2 cell culture on a device

# HepG2 cells were dissociated with trypsin/EDTA (0.04%/0.03%[v/v]) solution for 3 min at 37 °C and harvested from the dish. The cells were diluted with DMEM supplemented with 10% (v/v) FBS, 1% (v/v) nonessential amino acids, and 1% (v/v) penicillin/streptomycin, centrifuged at 200 ×*g* for 3 min and resuspended to 2.00 × 10^5^ in DMEM supplemented with 10% (v/v) FBS, 1% (v/v) nonessential amino acids, and 1% (v/v) penicillin/streptomycin. A total of 30 mL chambers-1 resuspended solution was then applied to a Matrigel-coated culture chamber, and cultured in humidified incubator at 37 °C with 5% CO2 for 24 h without the application of mechanical forces. At the end of day 1, the medium was replaced with DMEM supplemented with 10% (v/v) FBS, 1% (v/v) nonessential amino acids, and 1% (v/v) penicillin/streptomycin and cultured without mechanical forces. The media was changed after 72 h.

## Albumin ELISA

To measure albumin secretion using the Human Albumin EIA kit (TaKaRa), 20 μL of medium was collected from each well and preserved at -20 °C. The medium was diluted with the sample diluent solution at a 2:3 ratio. The diluted medium was introduced to a 96-well microplate coated with human albumin antibody and incubated at 22 °C for 2 h. The reacted solution was removed, and the wells were washed with 100 μL PBS three times. After the PBS was discarded, 100 μL antibody-POD Conjugate, dissolved in 11 mL of distilled water, was added to each well, and incubated at 22 °C for 1 h. The solution was discarded, and the wells were washed four times with PBS. After washing, 100 μL substrate solution (3,3ʹ,5, 5ʹ-tetramethybenzidine solution, TMBZ) was added, and incubated at 22 °C for 15 min. Subsequently, 100 μL of Wash and Stop Solution of ELISA without Sulfuric Acid (TaKaRa) was added and mixed to stop the reaction. The absorbance was measured at 450 nm.

# Supplementary Figures

**
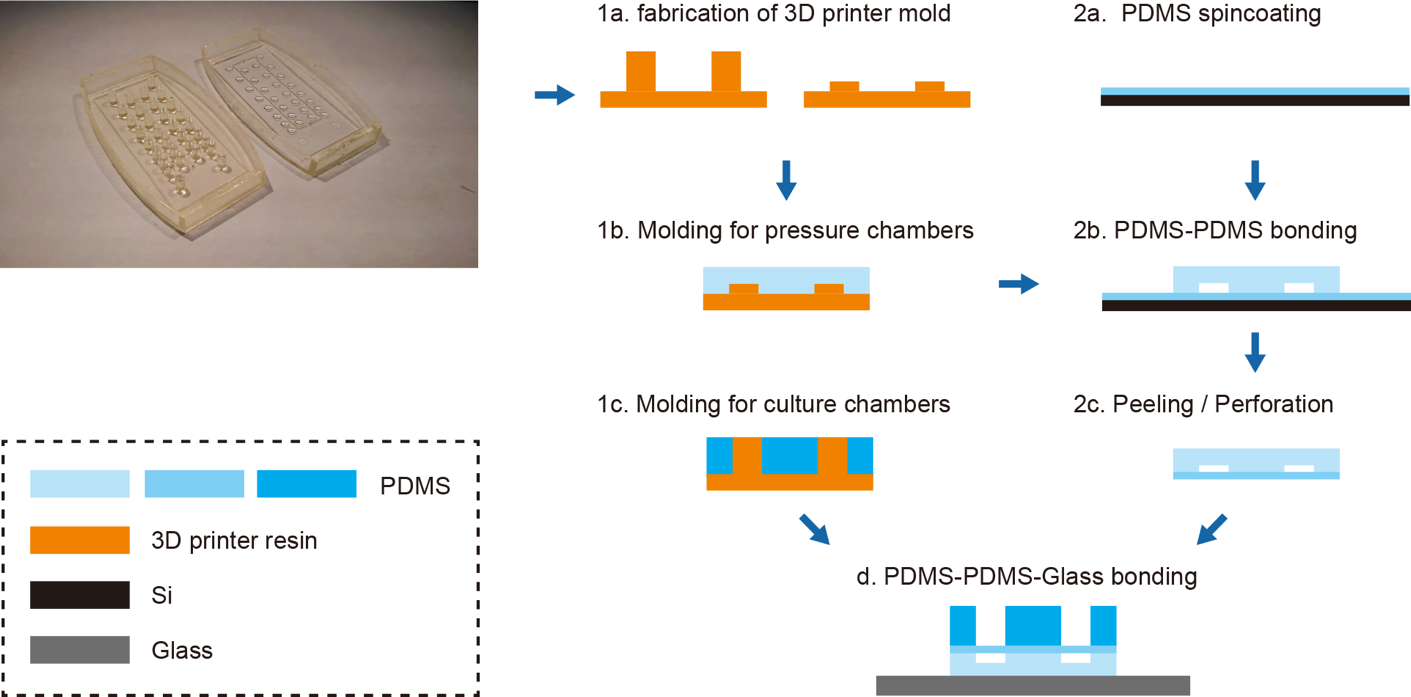
**

**Supplementary Figure S1.** Fabrication of a microfluidic device that recapitulates human embryonic heart beating. The device was fabricated as follows: (1a) For the top and bottom layer, the molds were fabricated with a 3D printer. (1b,c) PDMS was casted into the molds and cured at 80 °C for 16 h. (2a) PDMS was dropped on the silicon wafer, spin-coated at 500 rpm for 30 s, and cured at 80 °C for 10 min. (2b) The top layer and PDMS membrane were bonded together and baked at 80 °C at 1.5 h. (2c) The PDMS-PDMS forms were peeled off (d) and bonded to the top layer and glass.

**
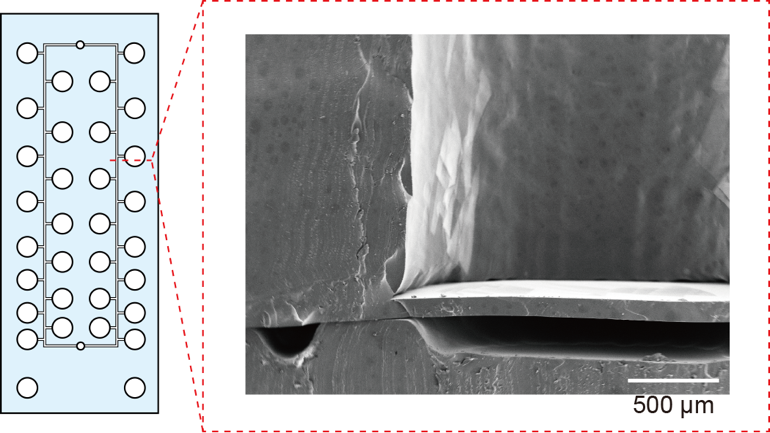
**

**Supplementary Figure S2.** Scanning electron micrograph of a cross-section of the microfluidic device. The micro channel and pressure chamber are indicated. Scale bar, 500 µm.

**
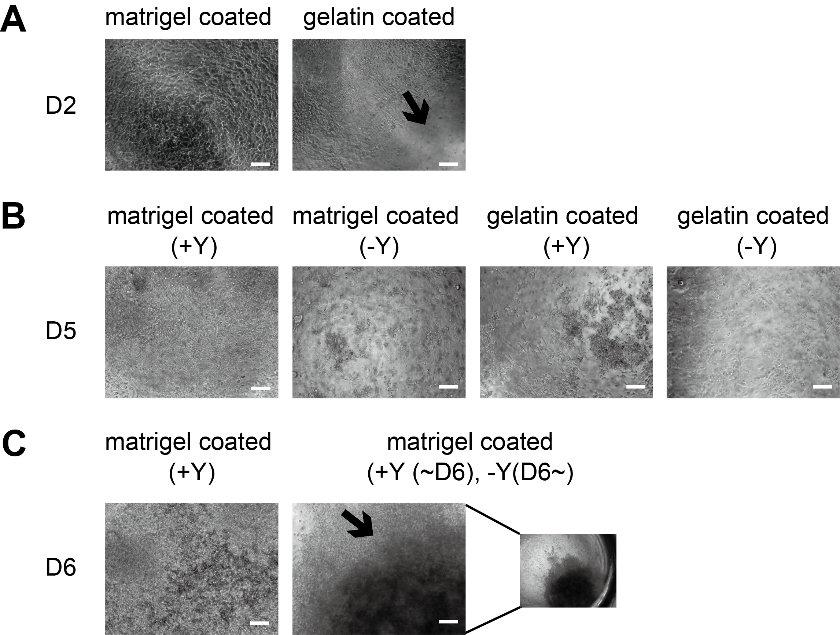
**

**Supplementary Figure S3.** Microphotograph of hPSCs-derived cells on the device. **A)** Detachment of cells on gelatin coated at day 2. Black arrow indicates the area of cell detachment. **B)** Detachment of most cells on Matrigel coated device without Y-27632 and gelatin coated device at day 5. **C)** The cells cultured with Y-27632 on the Matrigel coated device until day 6 detached and formed an aggregate within 30 min when medium with Y-27632 was changed to medium without Y-27632 at day 6. Black arrow indicates the cell aggregate. Y means Y-27632. Scale bars represent 100 μm.


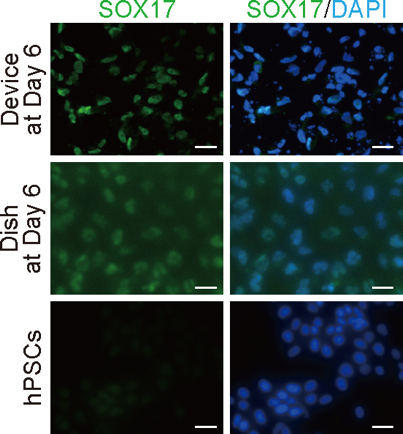


**Supplementary Figure S4.** Immunocytochemical analyses showing the expression of SOX17 in the indicated conditions. Nuclei were stained with DAPI. Scale bars represent 50 µm.

**
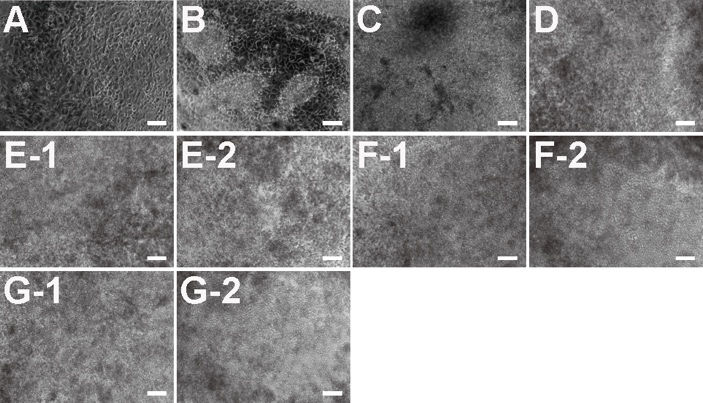
**

**Supplementary Figure S5.** Microphotograph of hPSCs-derived cells. **A)** hPSC-derived definitive endoderm (DE) cells at day 1. **B)** DE cells at day 4. **C)** Hepatic endoderm (HE) cells at day 8. **D)** HE-derived hepatoblasts at day 9 before stimulation. **E-1**) Cells without stimulation at day 10. **E-2**) Cells with stimulation (45, 10.4) at day 10. **F-1**) Cells without stimulation at day 11. **F-2**) Cells with stimulation (45, 10.4) at day 11. Scale bars represent 100 μm. **G-1**) Cells without stimulation at day 12. **G-2**) Cells with stimulation (45, 10.4) at day 12. Scale bars represent 100 μm.


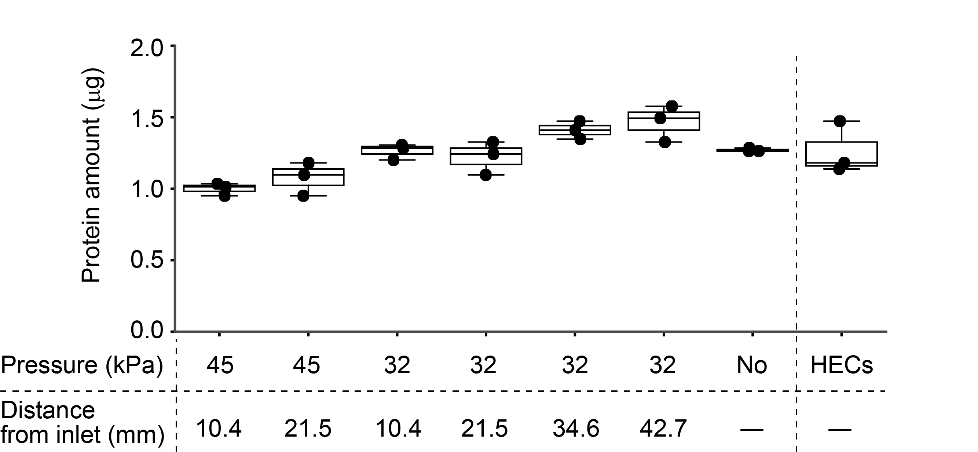


**Supplementary Figure S6.** Amount of protein in HECs and HBCs, used for bioluminescent CYP3A assay shown in **Figure 3C**. Boxplots were shown with max, median, and minimum values of three independent experiments.

**
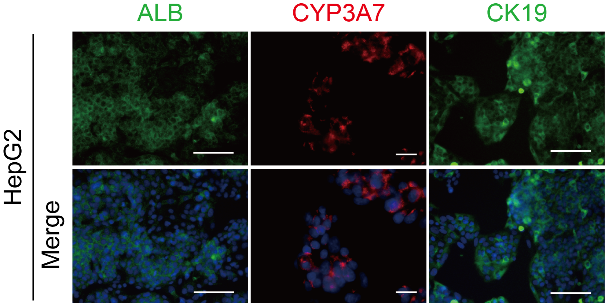
**

**Supplementary Figure S7.** Fluorescent micrographs of HepG2 cells as positive controls. Scale bars represent 100 μm and 50 μm.

**
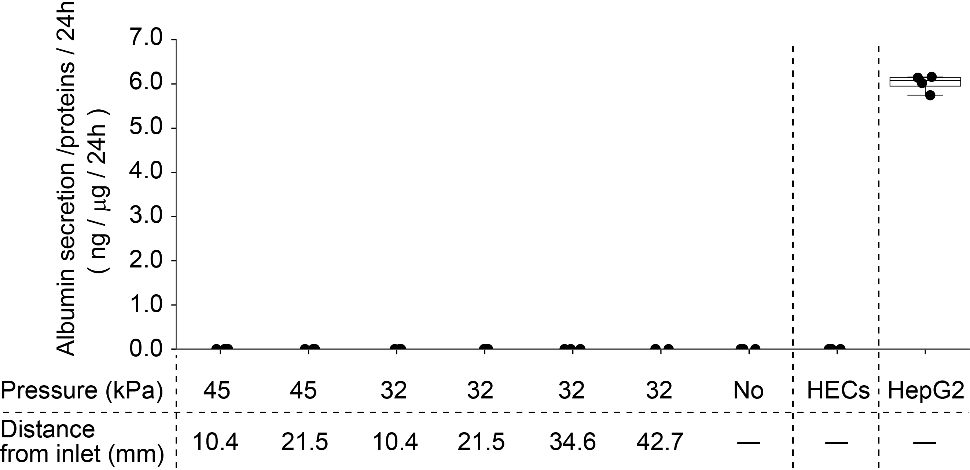
**

**Supplementary Figure S8.** Albumin secretion from HBCs, HECs and HepG2.

**3 Supplementary Videos**

**Supplementary Video S1.** Membrane oscillation at 45 kPa 0.2 Hz. The upper layer was sectioned, and the device was sputtered for visualization.

**Supplementary Video S2.** Mechanical stimulation to differentiate hepatic endoderm to hepatoblasts. (45, 10.4)
